# Characterization of the complete mitogenome of a land leech, *Haemadipsa crenata* Ngamprasertwong (Arhynchobdellida: Haemadipsidae)

**DOI:** 10.1080/23802359.2021.1939180

**Published:** 2021-06-21

**Authors:** Mo Wang, Xiangrong Tong, Yuan Su, Fanming Meng, Zichao Liu

**Affiliations:** a Engineering Research Center for Exploitation and Utilization of Leech Resources in Universities of Yunnan Province, College of Agriculture and Life Sciences, Kunming University, Kunming, China; b Key Laboratory for Conserving Wildlife with Small Populations in Yunnan, Faculty of Biodiversity Conservation, Southwest Forestry University, Kunming, Yunnan, China; c Department of Medical Parasitology, School of Basic Medical Sciences, Central South University, Changsha, China

**Keywords:** Mitogenome, land leech, *Haemadipsa crenata*, Haemadipsidae, blood feeding

## Abstract

Land leeches of genus *Haemadipsa* (Family Haemadipsidae) are widely distributed in South East Asia. *Haemadipsa crenata* Ngamprasertwong is a blood-feeding species firstly reported from Thailand. A complete mitochondrial genome of *H. crenata* was characterized in this study for further genetic exploration on land leech. The reads were assembled into a circular mitogenome of 14,725 bp in length. The AT content of H. crenata mitogenome is 76.79%. The annotated mitogenome contains 22 tRNAs, 2 rRNAs, and 13 protein-coding genes (PCGs), and the structure of PCG open reading frames was confirmed. Finally, the phylogenetic relationship of *H. crenata* and other leech species were reconstructed using mitogenomes.

Leeches (Annelida: Hirudinea) are a type of hermaphroditic and carnivorous worms with a constant number of segments and two distinctive locomotive suckers. Land leeches of family Haemadipsidae are distributed in some tropical areas of Asia and Australia (Borda et al. 2008). According to previous records, genus *Haemadipsa* (Haemadipsidae) is prevalent in Southeast Asia (Ngamprasertwong and Panha 2007). *Haemadipsa crenata* was first identified and described as a novel species in Thailand in 2007 (Ngamprasertwong and Panha 2007). The previously published studies of phylogenetic relationship between land leech species, including *Haemadipsa crenata*, were based on short barcode sequences like 18S, 28S or COI (Siddall and Burreson 1998; Borda and Siddall 2010; Schnell et al. 2018). Therefore, the phylogenetic relationship of land leech species remains worth of further exploration. Here, a complete mitogenome of the land leech *H. crenata* was presented. This mitogenome should facilitate further study on this issue.

The living *H. crenata* samples were captured from Mengsong Village, Menglong Town, Jinghong City, Yunnan Province, China (N21°53′, E100°63′). The leeches were deposited into pure ethyl alcohol and brought back to the lab. The jaw of leech was used for raw DNA materials extraction using CTAB method following a previous study (Skevington and Yeates 2000). The library construction and sequencing procedure followed the standard protocol of Illumina (Illumina Inc., San Diego, CA, USA). Then, the qualified DNA library (PE150) was sequenced on Illumina NovaSeq platform.

In total, 4.5 Gb data was generated and deposited into the NCBI database (BioProject: PRJNA695059, GenBank: MW711186). Voucher specimen was labeled with a unique serial number (CSU-KMU-MG20201101-1), then deposited into herbarium of department of College of Agriculture and Life Sciences, Kunming University. The cleaned reads were assembled into a 14,725-bp complete mitochondrial genome using SPAdes (Bankevich et al. 2012). In total, 13 protein coding-genes were identified from the mitogenome annotated with Mitochondrial Genome annotation (MITOS2) (Bernt et al. 2013) and open reading frames of each gene were confirmed by ORFfinder (https://www.ncbi.nlm.nih.gov/orffinder/). The tRNA secondary structures were compared with other leech tRNA sequences. 2 rRNA genes were also annotated in leech genome. Before the phylogenetic analysis, the mito-genome sequence of *H. crenata* was aligned with mtDNA sequences of 13 other leech species from the GenBank using the ClusterW (gap opening penalty of 30) embedded in the MEGA 7 software. Phylogenetic relationship was analyzed based on 13 mitogenomic protein coding sequences of *H. crenata* and other leeches using maximum likelihood (ML) method in MEGA 7 software with bootstrap of 1000 bootstrap replicates (Kumar et al. 2016). *Ozobranchus jantseanus* and *Zeylanicobdella arugamensis* were rooted as outgroups (Figure 1). The phylogenetic tree indicated that *H. crenata r*epresents a basal branch separated from the other Hirudinea species, and the relationship between those Hirudinea leeches was similar to previous researches (Nikitina et al. 2016; Liu et al. 2017).

**Figure 1.**
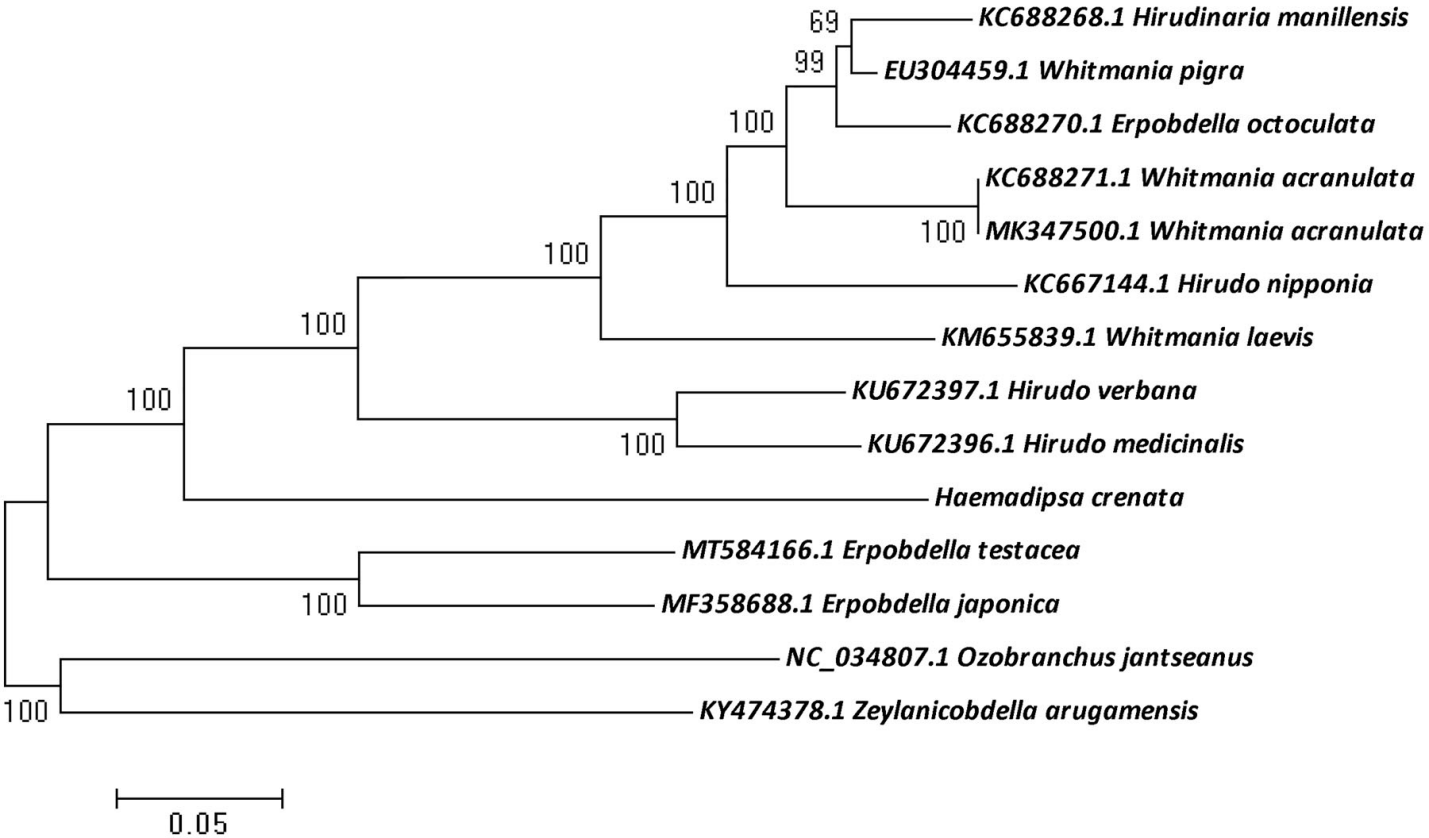
Phylogenetic tree of of H. crenata with other leeches using maximum likelihood (ML) method. The MEGA 7 software was used with bootstrap of 1000 bootstrap replicates.

## Data Availability

The data that support the findings of this study are now available. (https://www.ncbi.nlm.nih.gov/sra/PRJNA695059)
